# Surgical Risk on Patients with Coagulopathies: Guidelines on Hemophiliac Patients for Oro-Maxillofacial Surgery

**DOI:** 10.3390/ijerph16081386

**Published:** 2019-04-17

**Authors:** Luigi Laino, Marco Cicciù, Luca Fiorillo, Salvatore Crimi, Alberto Bianchi, Giulia Amoroso, Ines Paola Monte, Alan Scott Herford, Gabriele Cervino

**Affiliations:** 1 Multidisciplinary Department of Medical-Surgical and Odontostomatological Specialties, University of Campania “Luigi Vanvitelli”, 80100 Napoli, Italy; luigi.laino@unicampania.it (L.L.); lfiorillo@unime.it (L.F.); 2 Department of Biomedical and Dental Sciences and Morphological and Functional Imaging, Messina University, 98100 Messina (ME), Italy; amoroso.giulia@hotmail.it (G.A.); gcervino@unime.it (G.C.); 3 Department of Biomedical and Surgical and Biomedical Sciences Catania University, 95125 Catania (CT), Italy; torecrimi@gmail.com (S.C.); alberto.bianchi@unict.it (A.B.); 4 Department of General Surgery and Medical-Surgery Specialties, University of Catania, 95125 Catania (CT), Italy; inemonte@unict.it; 5 Department of Cardio-Thorax-Vascular and Transplant, A.O.U. Policlinico Catania, 95125 Catania (CT), Italy; 6 Department of Maxillofacial Surgery, Loma Linda University, Loma Linda, CA 92350, USA; aherford@llu.edu

**Keywords:** haemophilia, surgery, maxillo-facial, oral, coagulopathies, guidelines

## Abstract

Background: Haemophilia is a disease of genetic origin, which causes a defect in blood coagulation. Under normal conditions, in the case of leakage from the blood vessels, the blood forms a clot that reduces or blocks the bleeding. This process involves the activation of several plasma proteins in a cascade-like species. Two of these proteins, produced in the liver, factor VIII and factor IX, are deficient or present a functional defect in people with haemophilia. Because of this deficit, the haemophiliacs easily suffer external and internal bleeding. Surgical treatment of these patients is to be observed, and often their treatment is delayed due to unclear guidelines and risks in treating these patients. The aim is to provide clear guidelines in the case of surgical treatment of these patients. Methods: In this study, we have considered all the guidelines that refer to the gold-maxillofacial surgery, focusing on the literature of the last 10 years. Results: Surely, this collection of guidelines will favor the choice of the clinician towards safer and predictable protocols. This study does not want to create a guideline but evaluates the literature of the last 10 years, and highlights the latest for the treatment of these patients., with the aim of informing the pathology and at the same time making the surgical maneuvers safer. Conclusions: Despite the research of literature has produced few results, it was nevertheless possible to draw up a guideline thanks to additional information extrapolated from textbooks and other scientific articles. According to the guidelines, it is possible to proceed to the treatment of these patients, if with appropriate therapy in a safe and risk-free manner.

## 1. Introduction

Haemophilias are hereditary coagulopathies whose basic anomaly consists of the quantitative or qualitative alteration of one or more plasma proteins in the coagulation system. The entities most frequent nosological diseases are represented by: haemophilia A, haemophilia B and angioemophilia of von Willebrand. Haemophilia A has an incidence estimated at 1: 20,000–100,000 male births per year, of which the 30% of cases without familiarity (largely attributable to new mutations). It is a hereditary recessive coagulopathy linked to the X chromosome, in which the male is ill and has variously reduced plasma levels, while the female is usually a healthy carrier. The haemostatic defect derives from the lack of factor VIII to which follows a reduced generation of thrombin through the way intrinsic coagulation. The severity of the clinical picture is related to the degree of deficiency: a normal haemostasis requires a residual activity of >25%; the majority of patients have levels below 5% and have spontaneous symptoms and complications [1,2]. Inhibitors are most commonly encountered in people with severe hemophilia A (overall 25–40% lifetime risk) compared to those with moderate/mild hemophilia A (overall 5–15% lifetime risk). The symptomatology is represented by spontaneous haemorrhages or in relation to minor traumas (haematomas, haemarthrosis, arthropathies, gingivorrhagia, etc.). Up to 35% of severe haemophiliacs, antibodies of the IgG class (inhibitor of VIII inhibitors) appear to decrease the efficacy of substitution therapy and make the problem difficult treatment. Haemophilia B consists of factor IX deficiency and von Willebrand angioemophilia in a joint protein defect plasma of the coagulation cascade and vascular wall; the clinical picture is superimposable and requires replacement therapy with blood products [3,4,5]. Surgical medical treatment on these patients has the same demands as healthy patients, but in these patients it is necessary to evaluate the systemic conditions and therefore a difficulty in their approach. Surely, the surgeon will need advice from a haematologist to know if the clinical conditions of his patient allow a surgical intervention at that time or if it is necessary to carry out pharmacological manoeuvres. The protocols to follow are often complex and unclear; it is the purpose of this study to be able to clarify this topic [6,7,8,9,10].

## 2. Material and Methods

### 2.1. Focus Question

The following focus question was developed according to the population, intervention, comparison, and outcome (PICO) study design:

- What are the surgical guidelines for patients with coagulopathies, especially those with haemophiliacs?

### 2.2. Information Sources

The search strategy incorporated examinations of electronic databases, supplemented by hand searches. A search of four electronic databases, including Ovid MEDLINE, PubMed, EMBASE, and Dentistry and Oral Sciences Source, Biomaterials source for relevant studies published in the English language from January 2009 to January 2019 was carried out.

A hand search was also performed in Internal Medicine and Haematology journals. The search was limited to English language articles. A hand search of the reference lists in the articles retrieved was carried out to source additional relevant publications and to improve the sensitivity of the search.

### 2.3. Search

The keywords used in the search of the selected electronic databases included the following: “haemophiliac” OR “coagulopathies” AND “surgical” OR “oral” AND “guidelines”.

The choice of keywords was intended to collect and to record as much relevant data as possible without relying on electronic means alone to refine the search results.

### 2.4. Selection of Studies

Two researcher of University of Messina and University of Naples, singularly analysed the obtaining papers in order to select inclusion and exclusion criteria as follows. Reviewers compared decisions and resolved differences through comparing the manuscripts. For the stage of reviewing of full-text articles, a complete independent dual revision was performed.

### 2.5. Types of Selected Manuscripts

The review included studies published in the English language. Letters and Editorials were excluded.

### 2.6. Types of Studies

The review included all human studies and literature review published between January 2009 and January 2019, on haemophiliac patients undergoing to maxilla-facial district surgery.

### 2.7. Inclusion and Exclusion Criteria

The full text of all studies of possible relevance was obtained for assessment against the following inclusion criteria:

- Haemophiliac patients.
- Surgery and haemophiliac patients.
- Surgery on patients with coagulopathies.

The applied exclusion criteria for studies were as follows:

- Studies involving patients with other specific diseases, immunologic disorders, uncontrolled diabetes mellitus, osteoporosis, or systemic conditions.
- Not enough information regarding the selected topic.
- Surgeon guidelines not for dentistry or maxillofacial fields.
- Articles published prior to 1 January 2009.
- No access to the title and abstract in English language.

### 2.8. Sequential Search Strategy

After the first literature analysis, all article titles were screened to exclude irrelevant publications, case reports and the no English language publications. Then, researches were not selected based on data obtained from screening the abstracts. The final stage of screening involved reading the full texts to confirm each study’s eligibility, based on the inclusion and exclusion criteria.

### 2.9. Data Extraction

The data were independently extracted from studies in the form of variables, according to the aims and themes of the topic.

### 2.10. Risk of Bias Assessment

This type of work brings together all the studies in the literature in the last ten years presenting a technique for nanosurface realization and analyzing their biological interaction. The risk of bias in this case is present because each author convinced of his own study could have influenced the results. For this reason, the works taken into consideration and all those with a high risk of bias or with conflicts of interest have been carefully evaluated.

## 3. Results

### Study Selection

**Figure 1:**
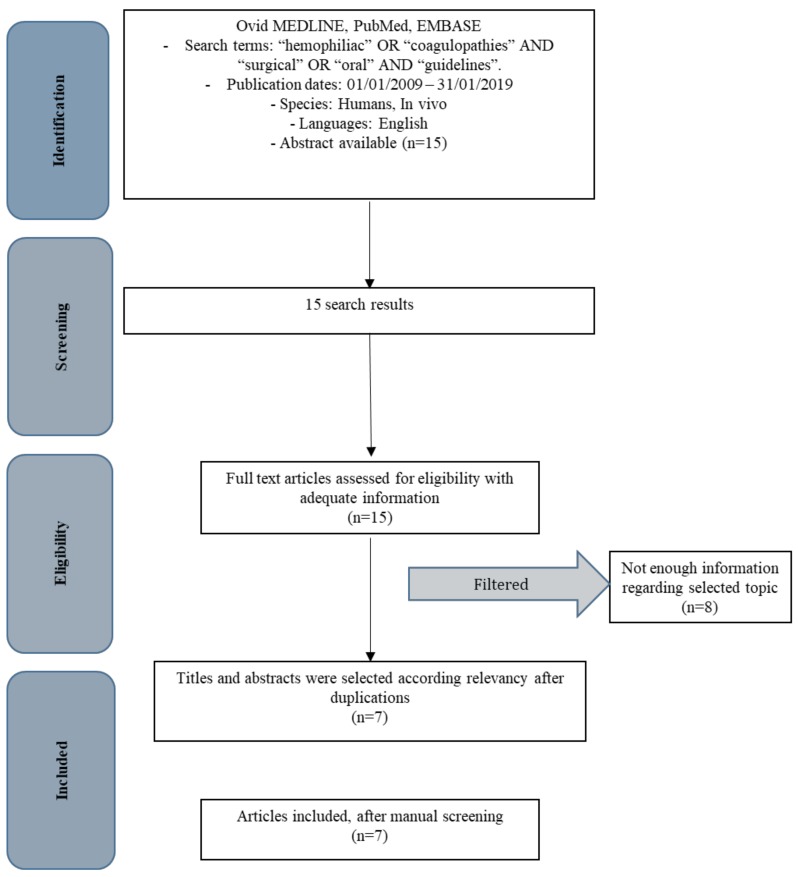
PRISMA flow diagram.

Perspective study and data extraction were performed according to PRISMA flow diagram (Figure 1). The initial electronic and hand search retrieved 20 citations. 5 papers were excluded because were excluded because pubblished before 1 January 2009. Out 111 results, 8 were excluded because are not suitable with the research. 

## 4. Discussion

### 4.1. Short Description of the Pathology

As already mentioned in the introduction, haemophilia is a recessive hereditary disorder that causes a serious deficiency in blood coagulation due to the total or partial lack of factor VIII (i.e., haeemophilia A) or of coagulation factor IX (The rarer is the factor IX (e.B), given by the total or partial lack of the XI factor. The hemophilic subject may be affected by spontaneous bleeding, or caused by trauma, these with outcomes that are more serious. In haemophilia type A the deficit can be quantitatively variable, so as to induce different clinical pictures: with functional levels of factor VIII up to 2% (i.e., A severe) haematomas are frequent (or the risk of serious bleeding, such as those cerebral) and, in childhood, the spontaneous hemarthrosis (hemorrhages of the joint cavities); with factor VIII levels ranging from 2 to 5% (i.e., A to moderate), hemarthrosis are rare and bleeding occurs only after surgery.

In haemophilia type B, the genetic anomaly determines a functional factor IX deficiency, which corresponds to a deficiency of the antigenic part in 70% of cases, and of only the functional part in 30%. Substitutive therapy rarely involves the infusion of prothrombin complex concentrates, the factor IX or VFIX is used frequently. Factor IX (e.B) (Haemophilia C) is even more rare and the defect, which affects the factor XI, is transmitted as an autosomal recessive deficit. The manifestations are more rare and milder than the previous defects: there are bleeding after surgery and after important traumas. Von Willebrand’s disease (VWD) is an inherited haemorrhagic disease caused by a genetic defect that determines a quantitative structural or functional anomaly of the Willebrand factor (VWF). The disease is classified into two main groups, based on the VWF deficiency: the form with a partial quantitative defect (type 1) or total (type 3), and the form with a qualitative defect (type 2). The clinical symptomatology includes the bleeding, of variable entity, that can arise spontaneously or during the use of invasive techniques. Bleeding abnormalities are usually characterized by muco-cutaneous haemorrhages [1,2,11,12].

Currently, the treatment of haemophilia occurs through the administration of the drug (hemoderivative or recombinant) containing the deficient coagulation factor. The two main therapeutic regimens for haemophilia are “on demand” therapy (when needed, i.e., at the time of bleeding) and prophylaxis, which instead involves the constant administration of the deficiency factor to prevent serious bleeding and protect patients. These are infusions that must be done about three times a week. For several months, recombinant drugs with a prolonged half-life are also available for prophylaxis therapy, which entail numerous advantages, allowing a lower number of infusions with equal protection for patients. In the future, gene therapy may be available for haemophilia [13,14,15].

### 4.2. Perioperative Risk and Surgery Manouvers

Perioperative risk surgical interventions and the same injections of anesthetic (both intramucose or truncular) may cause prolonged bleeding that can not be controlled with local measures and may cause serious consequences: hemorrhage with anemia until hypovolaemic shock [14]; Haematoma conspicuous up to the obstruction of the respiratory tract. An additional risk is the bacterial superinfection of a haematoma formed as a result of oral surgery [11]. Surgical maneuvers on these patients always expose them to a risk of bleeding, but the literature informs us that the patient’s life is unlikely to be at risk. For example, in the case of haemartis, very common complications of this pathology, surgery is now quite advanced, both in the diagnostic phase and in the therapeutic phase [4,15,16]. The damage in the joint, and the difficulties in the movement in this case require surgical, treatment and rehabilitation [4,17,18]. As for the oral district, surgical manoeuvres are often not advanced or complex, to reduce the operative risk. These may include the surgical removal of oral lesions or the extraction of non-recoverable dental elements with conventional conservative therapy, or with subgingival caries [19,20,21]. In any case, the extractive surgical maneuvers provide closure of the flaps for the first intention and techniques of socket preservation or socket shield so as to limit the postoperative bleeding and contain the clot [22,23]. Even a treatment such as scaling, or peri-implant surgery can expose the patient to bleeding. it is therefore necessary in these patients to maintain an accurate oral hygiene, so as to contain plaque accumulation, and inflammation of the soft tissues, not exposing the tissues to spontaneous bleeding or not [24,25].

### 4.3. Surgical Guidelines

Surgical treatment should be referred to hospital facilities and it is always necessary to request haematological advice to establish the severity of haemophilia, the presence of inhibitor VIII, the type of replacement therapy and the dosage, according to Table 1. Patients, also with severe haemophilia, but without inhibitor VIII, they can undergo most oral surgery after replacement therapy. NSAID prescription is contraindicated for decreased platelet activity and consequent increased risk of bleeding; Paracetamol and codeine can be used. In patients with mild-to-moderate haemophilia, it is preferable to use local intramucosal anesthesia and avoid intramuscular injections (truncular anesthesia); in patients with haemophilia, replacement therapy should be performed prior to any local anesthesia and/or bleeding treatment. 1 unit of factor VIII concentrate per kg of weight will increase plasma activity by about 2%. For major surgery, keep the levels of factor VIII >50% (Interventions with a high risk of bleeding >80%), postoperatively >30% for at least 10 days. The adequacy of the administered dose should be verified by dosage of factor VIII before and after administration, or in the impossibility of obtaining the specific dosage, by measuring, at the same time, the aPTT, which must normalize. It is necessary to ensure that the concentrate is completely dissolved and should be administered as soon as possible, and in any case within 30 min of reconstitution, in a slow intravenous bolus (5–10 min), without using glass infusion devices, in any case wait 1–2 hours for optimal effect. Simple extractions in patients with mild haemophilia A can be performed by administering desmopressin for nasal sprays (4–5 mg/kg in 50 mL of physiological in 15 min before surgery) and antifibrinolytics (amino-caproic acid 100 mg/kg per or every 6 h for 3–5 days or tranexamic acid). Replacement therapy before an oral surgery must guarantee a circulating factor VIII level of 50%. It is calculated an increase of activity equal to 1.5–2 times for each IU/kg body weight administered for 8–12 h (in practice 1UI increases the circulating level of 2%). The average dosage used consists of a single dose of 25–30 IU immediately before anaesthesia, followed by 10–20 IU/kg (50–75% of the initial dose) every 12 h on the following days in the case risk of bleeding assess the possibility of antibiotic therapy to prevent clot infection, however in these cases patients cannot coagulate without a correct protocol [3,7,14,25,26,27,28].

### 4.4. Studies Discussion

Planning elective surgery in people with haemophilia is always a challenge for the clinician. Haematology consultations are often requested and in many cases, the therapies, especially in the case of pain, are based only on a pharmacological phase that is repeated without ever reaching surgical therapy. Despite this, however, the guidelines and studies examined below are clear. According to Escobar et al., the planning phase should ensure that surgery proceeds without incident in a multidisciplinary surgery team, and it is very important that postoperative rehabilitation begin soon after surgery. The importance according to these authors therefore, lies entirely in the correct planning of the intervention, with a multidisciplinary team ready to handle any kind of complication [29]. In another study published in Evidence Based Dentistry in 2017, authors say that there is no clear indication to alter current practice utilising antifibrinolytic therapy to manage patients with haemophilia. According to this study, in fact, the guidelines evaluated up to now, would be correct and may still be followed in patients who go in for surgical treatment in the oro-maxillofacial district [30]. According to Zulfikar et al., the biggest challenge in patients with haemophilia is to perform surgical procedures because of bleeding. No deaths or life-threatening bleeding occurred during oral surgery in these patients. The results indicate that surgery can be safely performed by providing adequate and timely haemostasis and peri and postoperative bleeding complications are rare [31]. Coppola et al. evaluated 16 clinical trials, of these only four were eligible for inclusion with 112 participants. These trials compared the use of a different type and regimen of antifibrinolytic agents. These trials showed the reduction of blood loss if receiving antifibrinolytic. However, there is not sufficient evidence from randomized controlled trials to assess the most effective and safe haemostatic treatment to prevent bleeding in people with haemophilia [32]. Davis et al. in 2013 evaluated the bleeding risk during an invasive diagnostic procedure like gastro-intestinal endoscopy. Authors suggest that the use of tranexamic acid during gastroscopies, colonoscopies, sigmoidoscopies may be a safe approach for mild or moderate bleeding disorders [33]. An important study of Khaliavina et al. on dental care in haemophilia patients offers a guideline for surgery [34]. Gill et al. evaluated the surgery risks on von Willebrand disease patients, the results in this study indicate that von Willebrand factor and factor VIII concentrate (VWF/FVIII) is safe and effective in the prevention of excessive bleeding [35]. The surgical manoeuvres of these patients may be different, certainly, unlike patients who take antiplatelet drugs, they do not coagulate [36], even with the passage of time and with normal haemostatic manoeuvres, especially in severe cases. These patients may require complex surgeries, especially when there are oral lesions, or abnormalities [36]. Surely, the clinician will try to avoid unnecessary maneuvers, such as fine bone regenerative techniques for implant-prosthetic rehabilitations [36,37,38,39,40]. In the case of mild haemophilia it is necessary to request a hematological consultation and it is only possible to perform conservative treatments; the amount of blood factor VIII is always evaluated, which in the case of mild haemophilia is between 5% and 40% (usually the diagnosis is occasional). In the case of moderate haemophilia, with factor VIII between 1–5%, all the therapies that expose the patient to risk of bleeding (dental hygiene, deep conservative therapy, surgery), should be performed in a day hospital regime. In the case of severe haemophilia with factor VIII < 1% a replacement therapy with factor VIII concentrates must be performed; in this case the patients also have spontaneous bleeding, it is good that the patients are treated in specialized structures. In case of severe haemophilia with inhibitor VIII, factor VIII and immunosuppressants should be used as therapy, and surgery should be performed only if strictly necessary in specialized facilities. By analysing all the information, therefore, it is possible to draw up guidelines that affect the different forms of haemophilia and von Willebrand’s disease [41]. Even the psychological component, in these patients who discover their pathology as children, is very important. The haemophiliac patient of the new millennium is faced with conditions and life prospects very different from previous decades; this is due, as is known, to the considerable advantages obtained thanks to the prophylactic medical therapy that has allowed hemophilic to reduce the bleeding episodes and damages caused by arthropathy. In this sense, haemophilia has been able to broaden its horizons in search of a better quality and life expectancy, and it is in this context that the need to introduce rehabilitation treatment has developed. According Simurda et al., is successful avoidance of bleeding and improvement in quality of life after the prophylactic management with the administration of replacement clotting factor when compared with on-demand regimen in haemophiliacs. Prolonged half-life drugs, developed with FC fusion technology, produced with recombinant DNA technology in a line of human embryonic cells: ELOCTA (recombinant factor VIII, efmoroctocog alfa) for haemophilia A—and ALPROLIX (recombinant factor IX, eftrenonacog alfa) for haemophilia B. These are drugs made thanks to genetic engineering: the coagulation factor is melted in the FC portion of the specific immunoglobulin (a particular antibody): in this way it is possible to use a natural pathway for prolong the residence time of therapy in the body (half-life). furthermore, patients can continue the prophylactic treatment by reducing the number of weekly infusions without compromising the protection from bleeding episodes, all with a long-term impact on the state of the joints, adherence to therapy and quality of life. The reduction in the number of infusions is the requirement that our patients have always emphasized and now we have the concrete possibility of offering it, creating the conditions for an improvement in adherence to therapy with a significant positive impact on the quality of life of patients, with no differences about surgery protocols [14,42,43,44,45,46,47,48,49,50,51]. At last only 7 studies were included because on human science field (Table 2).

### 4.5. Limitations

Despite the limited scientific support available in the literature, this study is able to represent a guideline for the surgery of these patients. Surely, the patient must be framed and carefully evaluated even by haematological counselling. The guidelines analyzed in this study, and therefore proposed by the authors, should in any case be re-evaluated according to the new biomedical technologies. it is necessary to remember that in the course of the literature analysis, no guideline taken into consideration mentioned the use of biomaterials or other factors such as micro-crystallization collagen, anti-fibrinolytic and factor replacement. The guidelines, however, inform us of a low risk towards patients’ health.

## 5. Conclusions

This article is intended to be a report of the articles and guidelines in literature over the last 10 years regarding this topic. The results appear to be satisfactory and to offer surgeons’ risk-free guidelines. in any case, the patient must be considered with a multidisciplinary approach and be attentive during the intra and post-operative phases.

## Figures and Tables

**Table 1:** Surgical guidelines for haemophiliac patients.

Surgical Risks	Guidelines
Mild haemophilia	Request haematological advice Perform conservative therapies in external outpatient treatment Intramucosal anesthesia (plessica) Do not prescribe NSAIDs Demand surgical treatments for hospitals Desmopressin and antifibrinolytics for simple extractions
Factor VIII 5–40% Occasional diagnostic	
Moderate haemophilia	Surgical therapies in day care regimen or hospitalization Local haemostatic measures antifibrinolytic Replacement therapy (factor VIII concentrates plasma-derived or recombinant)
Factor VIII 1–5% Spontaneous haemorrhages, haematomas or hemartri	
Severe haemophilia	
Factor VIII <1% Early onset (childbirth, first attempts at walking) with severe spontaneous haemorrhages and permanent sequelae (recurrent hematria and articular ankylosis)	
Severe haemophilia with inhibitor VIII	Contraindicated surgical therapies to be applied only in the absence of therapeutic alternatives Necessary high-dose factor VIII dosages are needed Therapeutic alternatives consist of using factor VIII

**Table 2:** Haemophiliac guidelines from studies.

Author	Treatment	Aim
Escobar et al. [29]	• Evaluation of patient’s suitability for surgery • Agreement with patient/family on goals and realistic expectation of surgical outcome • Planning procedure Scheduling of surgery • Full assessment of patient’s physical condition • Surgery Management of unexpected bleeding events • Follow-up with patient • Discussion of postoperative rehabilitation • Dental checks for inflammation and tooth decay (risk of infection), including radiographic assessment of the dentition and mandible and maxilla	Multidisciplinary approach in people with haemophilia
Watterson et al. [30]	• Antifibrinolythic and dental extraction (Low quality evidence exists to support the use of adjuvant antifibrinolytic therapy)	Preventive and operative dentistry on patients with bleeding disorders
Zulfikar et al. [31]	• Perioperative and postoperative bleeding complications are rare	Surgery on von Willebrand patients
Coppola et al. [32]	(1) Replacement treatment (a) Duration from 5 to 10 days (2) Thromboembolic adverse events (a) Was no reported (3) Mortality (a) Were no participant deaths (4) Blood loss (a) During surgery (i) No blood loss by variations of haemoglobin levels (ii) In some cases blood transfusion is reported (b) After surgery (i) No blood loss by variations of haemoglobin levels (ii) In some cases blood transfusion is reported, with no results (5) Need for re-interventionRe-intervention was reported once because of an haematoma (6) Secondary outcomes of the study (a) Need for additional dosing of the drug. The total number of additional rFVIIa bolus injections beyond the two allowed by the protocol in any 24-h period was similar (b) Need for alternative haemostatic treatment Five participants with haemostatic failure on the assigned rFVIIa regimens were moved to alternative haemostatic therapy (two activated prothrombin complex concentrate, one porcine FVIII, one recombinant FVIII and one EACA) (c) Haemostatic effectiveness A higher rate of efficacy was reported in favour of the high dose group at post-operative assessments from day three to day five, with a more pronounced effect in major than in minor surgery (rFVIIa bolus). (d) Duration of replacement treatment Treatment with rFVIIa was 10 days (e) Concentrate consumption Consumptions is greater in the continuous infusion arm than in bolus treatment arm. (f) Thromboembolic adverse events Thrombosis was reported, adoption of venous thromboembolism prophylaxis is needed, mechanical manoeuvers are reported.	Preventing bleeding on haemophiliac or bleeding disorder patients
Davis et al. [33]	This pilot feasibility study suggests that the use of Tranexamic Acid, without prophylactic factor replacement or DDAVP preprocedure appears to be safe.	Tranexamic acid for prevention of bleeding in bleeding disorder during endoscopy
Khaliavina et al. [34]	(1) Dentistry (a) Careful use of saliva ejectors; (b) Careful removal of impressions; (c) Care in the placement of X-ray films, particularly in the sublingual region; (d) Protection of soft tissues during restorative treatment by using a rubber dam or applying yellow soft paraffin (vaseline^®^). (e) Restorative can be undertaken routinely providing care to mucosa (f) Orthodontic treatment can be used (g) Patients can use dentures (2) Anesthetic (NO HAEMOSTATIC COVER REQUIRED) (a) Buccal infiltration (b) Intra-papillary injections (c) Intraligamentary injections (3) Anesthetic (HAEMOSTATIC COVER REQUIRED) (a) Inferior dental block (b) Lingual infiltration (4) Surgery (a) Planning (i) Clinical and Radiographic exams (ii) Prophylactic cover required by patient (iii) Observe all patients after dental extractions, up to few hours (iv) Discuss with an haemophilia unit (v) Discuss the use of local haemostatic agents (fibrin glue, oxidized cellulose) (vi) Antibiotic prophylaxis (vii) Atraumatic treatment (b) Preoperative (i) Work in a healthy oral cavity, remove plaque, and use antibacterial mouthwash (ii) Consider using an antifibrinolytic agent (c) Intraoperative (i) Patients rinse with chlorhexidine for 2 min (ii) Atraumatical extraction (iii) Suture the socket (iv) Use local haemostatic measures (v) Protect the socket (d) Postoperative (i) No mouth rinsing for 24 h (ii) No smoking for 24 h (iii) Soft diet for 24 h (iv) Bo strenuous activities for 24 h (v) Analgesia (vi) Salt-water mothwashes (4 times/day for 7 days from 2nd day) (vii) Antibacterial mouthwash (viii) Emergency contact details to patient (e) Post-extraction haemorrhage (i) Use tranexamic acid (ii) Monitor blood pressure (iii) Contact haemophilia unit (iv) Fibrin glue (v) Splints (5) Oral Infections (a) Dental Infections (i) Antibiotic prophylaxis (b) Periodontal infection (i) Chlorexidine gluconate treatment (ii) Povidine-iodine treatment	Bleeding disorder and dentistry
Gill et al. [35]	Effective haemostasis with the use of VWF/FVIII concentrate treatment	Von Willebrand management with therapies
